# Serum INSL3 measured by LC-MS/MS in pubertal girls and in girls with precocious puberty during GnRH agonist treatment

**DOI:** 10.3389/fendo.2024.1404320

**Published:** 2024-07-01

**Authors:** Jakob Albrethsen, Casper P. Hagen, Anders Juul

**Affiliations:** ^1^ Department of Growth and Reproduction, Copenhagen University Hospital - Rigshospitalet, Copenhagen, Denmark; ^2^ International Centre for Research and Research Training in Endocrine Disruption of Male Reproduction and Child Health (EDMaRC), Rigshospitalet and University of Copenhagen, Copenhagen, Denmark; ^3^ Department of Clinical Medicine, University of Copenhagen, Copenhagen, Denmark

**Keywords:** INSL3, LC-MS/MS, puberty, girls, central precocious puberty

## Abstract

**Introduction:**

The peptide hormone Insulin-like Factor 3 (INSL3) is a biomarker of testicular Leydig cells in the male but is also expressed by the theca cells of the ovaries. With the advent of sensitive assays INSL3 can be quantified in female circulation, and we suggest that circulating INSL3 is a novel biomarker for pubertal development in girls. The aim of the study is to quantify INSL3 by LC-MS/MS in sera from normal girls during pubertal transition, and during gonadal suppression by GnRH agonist therapy in girls with central precocious puberty (CPP).

**Method:**

The sensitivity of an established LC-MS/MS-based method for serum INSL3 was improved by switching to a state-of-the-art triple quadruple mass spectrometer (Altis Plus, Thermo).

**Results:**

The limit of detection of the improved LC-MS/MS method for serum INSL3 was 0.01 ug/L (1.5 pM) and the inter-assay CV was < 12%. Serum INSL3 increased during the pubertal transition in healthy girls and changes correlated with the concomitant rise in other measured hormones. In some girls, but not all, INSL3, FSH, inhibin B and estradiol serum concentrations increased prior to first clinical signs of puberty. Serum INSL3 concentrations were increased at baseline in girls with CPP compared to prepubertal controls and decreased during treatment with GnRH agonist followed by a steep rise and normalization after cessation of treatment.

**Conclusion:**

The improved method allowed for quantification of INSL3 in longitudinally collected serum samples during pubertal transition in healthy girls as well as in girls with CPP before, during and after treatment with GnRH agonist. Future studies are needed to clarify if INSL3 in combination with other biomarkers enhances the predictive value of differentiating between premature thelarche and CPP.

## Introduction

The peptide hormone Insulin-like factor 3 (INSL3) is produced by the ovarian theca cells and the leydig cells of the testes. INSL3 targets the receptor Relaxin Family Peptide Receptor 2 (RXFP2) and is important for the proper testicular descent in the male fetus (1). The biological function as well as potential clinical use of INSL3 in girls remains to be elucidated.

Evaluation of ovarian function in girls suspected of precocious puberty includes assessment of secondary sexual characteristics (breast and pubic hair development), growth velocity and bone age. Further evaluation may include quantification of circulating concentrations of reproductive hormones, e.g. gonadotropins (LH and FSH) as well as specific ovarian hormones. More specifically, ovarian granulosa cells produce AMH, inhibin B and estradiol, whereas ovarian theca cells produce testosterone and androstendione (2). INSL3 is exclusively produced by ovarian theca cells from medium and large antral follicles as well as in corpora lutea (3, 4). Theca cells convert cholesterol to androgens which are further aromatized to estrogens by the neighbouring granulosa cells (5). As opposed to androgens which are also produced by the adrenals, INSL3 seems to be a specific marker of ovarian theca cell function. However, the physiological role of INSL3 in females is not fully elucidated. Insl3 -/- female mice vs wildtypes have similar numbers of follicles in different stages and unaffected estrous cycle and litter size. However, accelerated follicular atresia and involution of corpus luteum in INSL3 KO mice, suggest that INSL3 protects these structures from apoptosis (6, 7).

In adult women, circulating levels of INSL3 decrease with increasing age, and fluctuations during the menstrual cycle seem to reflect waves of FSH-induced antral follicles (2, 8). Undetectable serum INSL3 concentration after menopause support that circulating INSL3 is exclusively produced by the ovary (1). As a marker of theca cell activity, serum concentrations of INSL3 are elevated in patients with PCOS because of an abundant number of INSL3 producing ovarian follicles (2, 9, 10). In peripubertal girls, circulating INSL3 concentrations increase in late puberty, however levels vary substantially between individuals (2, 11).

In the present study we utilize a new MS-instrument allowing for improved sensitivity when applied in our previously developed LC-MS/MS method for quantification of serum INSL3 concentrations (12). We measure serum INSL3 in healthy peripubertal girls as well as in girls with central precocious puberty (CPP) before, during and after GnRHa treatment.

## Methods and materials

### Subjects and samples

The study is based on secondary analysis of blood samples collected in conjunction with two previous studies of healthy pubertal girls and of girls with CPP, respectively.

Healthy girls: A total of 78 longitudinally collected serum samples obtained from nine healthy Danish pubertal girls were selected from the Copenhagen Puberty Study (13). Detailed description of ethical permissions, sample collection and the participating girls, including definition of pubertal onset and serum AMH, FSH, LH, Inhibin B and Estradiol levels have previously been published for these samples (13–16). The girls were classified according to Tanner’s classification and pubertal onset was defined as advancement from B1 to ≥ B2 at the following examination. The nine girls included in the present study is a nested cohort of healthy girls; criteria for selection: multiple examinations prior to pubertal onset and through pubertal development including available blood samples. Serum INSL3 has previously been measured in the samples using immunoassay (13) and this data is used for method comparison in the present study.

CPP Girls: A total of 46 serum samples were selected from 15 Danish girls during GnRH agonist treatment. Detailed description of ethical permissions, sample collection, the participating girls and their treatment and serum AMH, FSH, LH, Inhibin B, Estradiol has previously been published for these samples (17, 18).

### Hormone analyses

For INSL3 analysis, serum was prepared as previously described (12). In brief, serum, calibrants and quality control (QC) samples (150 μL) were transferred to tubes (LoBind, Eppendorf), and sample reagent (1.5 M NH4 AC, 85% EtOH, with internal standard) was added (350 μL). The solution was mixed (600 × g for 10 s and 100 × g for 10 min) and left at −20 °C for minimum 1 h and centrifuged (12.700 × g, 10 min, 4 °C). The supernatant (450 μL) was collected and was first reduced by addition of 5 μL DTT (final conc. 1 μM, shaking for 30 min, 100 × g, 60 °C), and then alkylated by addition 5 μL IAM final conc. 2 μM, stored in the dark, 30 min room temperature). The solution was evaporated (nitrogen flow, 45 °C, 90 min) and the dried pellet was dissolved (120 μL, 50 mM NH4 HCO3 (pH 8)) and shaken (2 min, 100 × g, 60 °C) and centrifuged (12.700 × g, 10 min, 4 °C). The supernatant (120 μL) was transferred to vials for LC-MS/MS analysis. INSL3 samples were kept at 10 °C and injected (100 μL) using an HTS PAL autosampler (CTC Analytics AG, Switzerland). LC-MS/MS analysis was performed on an Aria 4 pumps UHPLC system with integrated Transcend TLX TurboFlow coupled to a triple quadrupole mass spectrometer (TSQ Altis plus, Thermo Scientific, USA) controlled by Aria MX 2.6 and Xcalibur 4.5 software (ThermoFinnigan, USA). The turboflow column was a Cyclone-P Turboflow column (proprietary stationary phase, 50 mm × 0.5 mm, Thermo) kept at 25 °C, and the analytical reverse phase-column was Kinetex (reverse phase C18, 50 mm × 2.1 mm, 2.6 μM, 100 Å, Phenomenex) with Security Guard Ultra (reverse phase C18 filter, 2.1 mm × 4 mm, 2.6 μM, Phenomenex) kept at 45 °C. Eluent A was 0.2% FA in MQ water, and eluent B was 100% ACN. The total runtime was 10.1 min with a solvent flow on the eluting side of 0.3 mL/min, and the gradient increased from 12% to 30% solvent B. The MS instrument was equipped with a heated electrospray ionization probe operated in positive mode. The spray voltage was 3500 V. Sheath gas (nitrogen), aux gas and sweep gas was set at 42, 25 and 1 (arbitrary units), respectively. The ion transfer tube was kept at 350 °C, and the vaporizer temperature was 425 °C. A cycle time of 0.2 s was used, and resolution (FWHM) at both Q1 and Q3 was 0.6. Argon was used as collision gas at a pressure of 2 mTorr and Chrom filter set at 3 (arbitrary unit). Collision energies were 25 V and 30 V for the quantifier (m/z 1002.8 -> 279.1) and qualifier ions (m/z 1002.8- 439.1), respectively.

Measurement of AMH, FSH, LH, Inhibin B, testosterone, androstendione and Estradiol was performed as previously described (19, 20). In brief, serum AMH levels were determined using the Beckman Coulter enzyme immunometric assay (Immunotech, Beckman Coulter Ltd.) with a detection limit of 2.0 pmol/L and intra-assay and interassay coefficients of variations (CVs) of 7.8% and 11.3%, respectively. Serum FSH and LH levels were measured by time-resolved immunofluorometric assays (Delfia; PerkinElmer) with detection limits of 0.06 and 0.05 IU/L for FSH and LH, respectively. Intra-assay and interassay CVs were less than 5% in both gonadotropin assays. Inhibin B levels were measured by double-antibody immunometric assays (Serotec) with a detection limit of 20 pg/mL, and intra-assay and interassay CVs less than 16%. Serum testosterone and androstendione were measured by LC-MS/MS with a detection limit of 0.10 nmol/L (T) and 0.18 nmol/L (A), and with an inter-assay CVs < 12%. Estradiol levels were determined by RIA (Pantex) with a detection limit of 18 pmol/L and intra-assay and interassay CVs of 7.5% and 12.3%, respectively. Hormone measurements that fell below the assay LOD were adjusted to half the value of the respective LODs.

### LC-MS/MS method sensitivity and precision

The LC-MS/MS method for quantification of human serum INSL3 concentration was previously validated according to CLSI guidelines (21), including examination of sensitivity, precision, linearity, selectivity, stability, recovery, and method comparison with immunoassay (12). Here we introduce a modified method for serum INSL3 in which the original MS instrument (TSQ Vantage, Thermo) is substituted for a newer instrument (TSQ ALTIS plus, Thermo). The sensitivity of the improved method was examined by adding recombinant INSL3 (quantitated by amino acid analysis (see previous study for method validation details (12)) to a pool of serum collected from three post-menopausal women (commonly used as control matrix for both the INSL3 immunoassay and LC-MS/MS assay (1, 12)). Five replicates of the following nine concentrations were prepared individually and measured in one batch: 0 (blank), 0.005, 0.012, 0.017, 0.025, 0.05, 0.15, 0.5, 1.0 and 2.0 ug/L. The method was compared to serum INSL3 previously measured by immunoassay (LOD=0.01 ug/L) in the same 78 serum samples from healthy girls (11).

### Statistical methods

Means, standard deviations, CVs, and p-values (Student’s t-test for means, Student’s t-distribution) were calculated in Excel (Microsoft, 2016). Correlations between circulating levels of different hormones were assessed by Spearman correlation including all available data from the longitudinal dataset. LOD and LOQ was determined both according to the standard deviation only (LOD = (average for five blanks) + (5 x standard deviation of five blanks)) and by the slope (LOD = 3.3 x (standard deviation of five blanks)/(slope of the calibration curve)) and LOQ = (10 x (standard deviation of five blanks)/(slope of the calibration curve)).

## Results

### Sensitivity and precision of serum INSL3 on the TSQ ALTIS plus instrument

**Figure 1:**
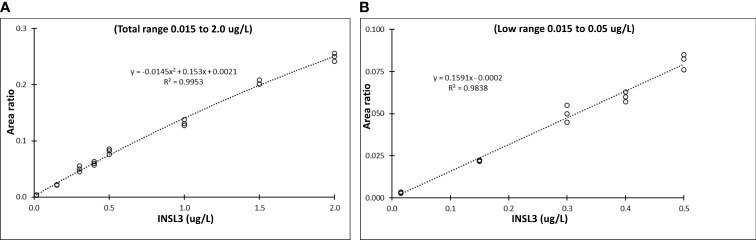
Sensitivity of serum INSL3 measurements with the TSQ Altis Plus Triple Quadrupole Mass Spectrometer (Thermo) **(A)** Nine-point calibration curve for serum INSL3 in the entire range from 0.015 to 2.0 ug/L with a quadratic fit. **(B)** Five-point calibration curve for serum INSL3 in the low range from 0.005 to 0.15 ug/L and with a linear fit.

By switching to a newer MS-instrument (TSQ ALTIS plus, Thermo) we improved the sensitivity with LC-MS/MS of serum INSL3. In addition to five calibration points in the established range of quantification (0.05, 0.15, 0.5, 1.0 and 2.0 ug/L) we examined four additional concentrations in the lower range (0.015, 0.15, 0.3 and 0.4 ug/L) and the resulting non-linear curve was best fitted to a quadratic equation (R2 = 0.99) Thermo Xcalibur Quan Browser (**Figure 1A**). First, we estimated the sensitivity based on the standard deviation and obtained an LOD = 0.006 ug/L. Next, the five lowest calibration points alone (0.005, 0.012, 0.017, 0.025, and 0.05 ug/L) demonstrated a linear fit (R2 = 0.98) and based on the slope we obtained an LOD = 0.01 ug/L. In addition, using the slope we obtained an LOQ = 0.03 ug/L (**Figure 1B**). In subsequent studies we decided on the sensitivity obtained using the slope of the linear fit in the low range (LOD = 0.01, corresponding to 1.5 pM). The method was introduced in a routine-like setting and the precision was estimated based on measurement of two QC samples: QC1 = 0.3 ug/L and QC2 = 1.0 ug/L. The QCs were measured 118 times in 48 batches over a period of 16 months, using the new instrument. The inter-assay precision for QC1 and QC2 was 11.8% and 8.1%, respectively.

### Serum INSL3 of healthy pubertal girls

**Figure 2:**
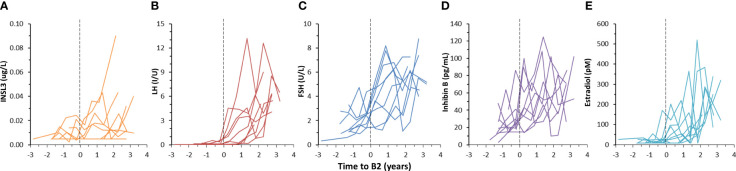
Longitudinal changes in serum INSL3 **(A)**, LH **(B)**, FSH **(C)**, Inhibin B **(D)** and estradiol **(E)** during puberty in nine girls. Pubertal onset is defined to occur at the midpoint between the two examinations where a girl progress from stage B1 to ≥ B2, according to the Tanner’s classification, and is indicated by the dotted vertical lines **(A–E)**.

**Figure 3:**
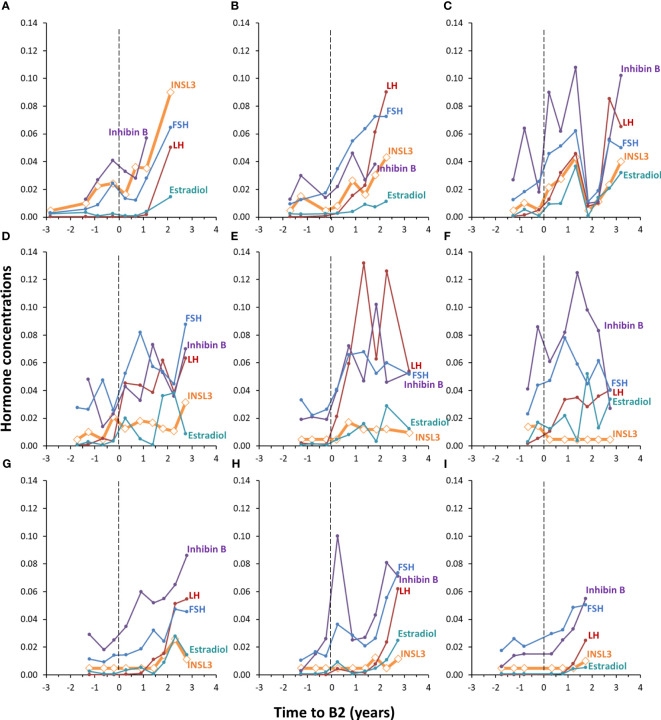
Individual longitudinal changes in serum INSL3 (orange), LH (red), FSH (blue), Inhibin B (purple) and estradiol (turquoise) during puberty in nine healthy girls **(A–I)**. The y-axis represents the concentration of INSL3 (ug/L). The concentrations of the other four hormones are adjusted to fit in the same range; LH (I/U) is divided by 100, FSH (U/L) is divided by 100, Inhibin B is divided by 1000 and estradiol (pM) is divided by 10000. Pubertal onset is indicated by the dotted vertical lines **(A–I)**.

The concentration of INSL3 showed an overall increasing trend through puberty in 78 serum samples longitudinally collected from nine girls (**Figures 2**, **3**). Of the 78 samples, 31 were below the LOD. Serum INSL3 was significantly increased (p < 0.001, t-test for means) in samples collected after puberty (average = 0.016 (0.015) ug/L (n = 50), as compared to before puberty (average = 0.008 (0.006) ug/L (n = 28)). One exception was a single girl where serum INSL3 was only detectable in two measurements before puberty and was below LOD in all samples collected after puberty (**Figure 3F**). For several girls, there was substantial and often parallel fluctuations in the studied hormones (see for example **Figure 3C**). Serum INSL3 levels correlated positively with LH (R=0.37, p<0.001), AMH (R=0.23, p<0.05), Inhibin B (R=0.27, p<0.02), estradiol (R=0.23, p<0.04), FSH (R=0.38, p<0.001), testosterone (R=0.43, p<0.001) and androstendione (R=0.33, p<0.003).

### Serum INSL3 in girls with central precocious puberty treated with GnRH agonist

**Figure 4:**
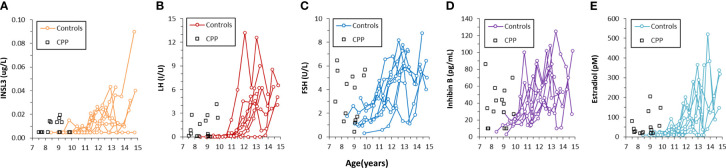
Concentration of INSL3 **(A)**, LH **(B)**, FSH **(C)**, Inhibin B **(D)** and estradiol **(E)** in serum longitudinally collected from nine healthy pubertal girls (colored lines) and in serum collected from 15 girls with central precocious puberty before the beginning of treatment (black squares).

**Figure 5:**
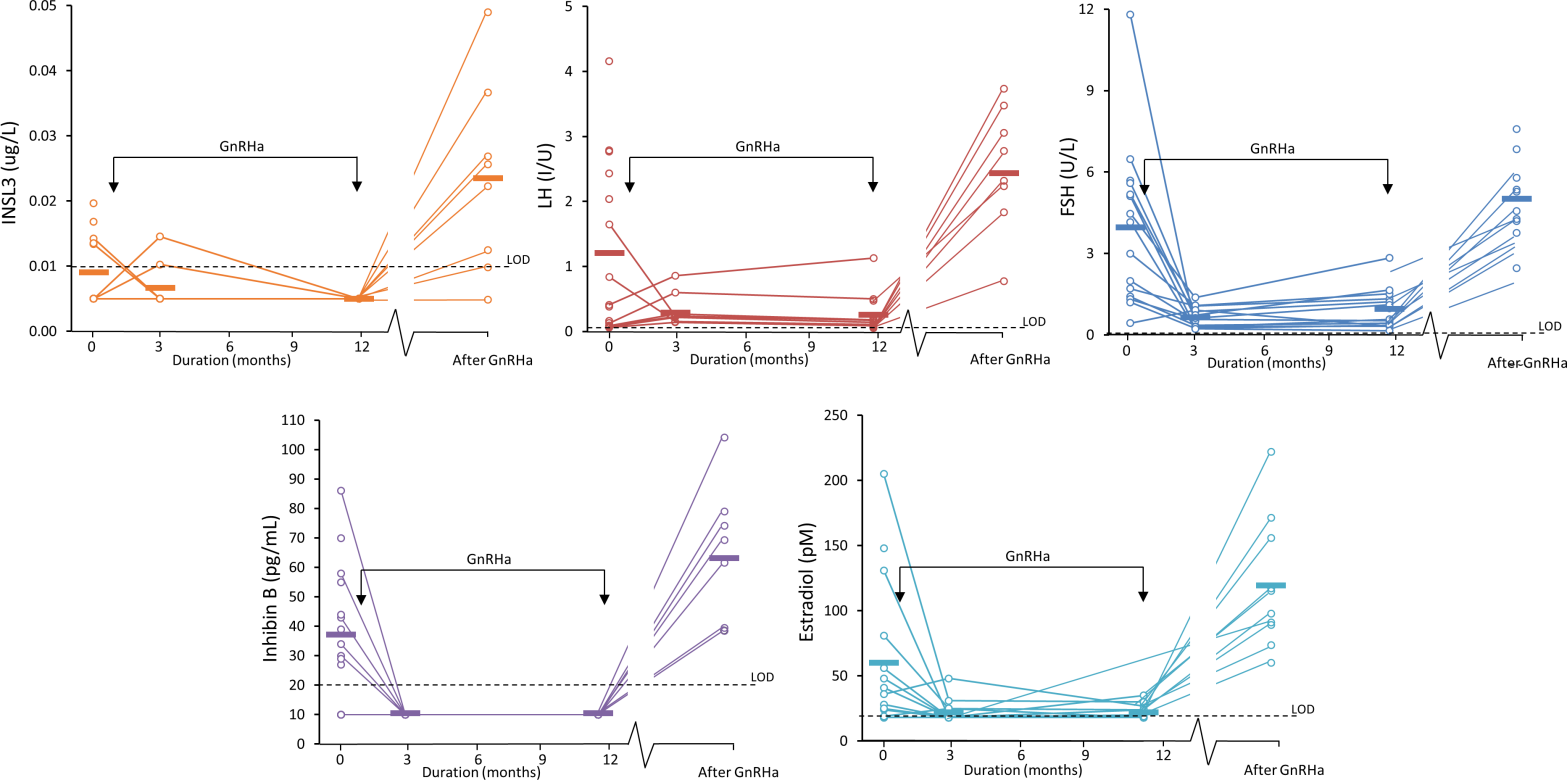
Serum INSL3 (orange), LH (red), FSH (blue), Inhibin B (purple) and estradiol (turquoise) measured in 15 girl patients during treatment for central precocious puberty (CPP). The girls were treated with GnRH agonist at the time indicated by the two arrows. The bars indicate the average concentration at each of the four sample collections: 0 months (before treatment/baseline), 3, and 12 months (during treatment and 36 months) after treatment.

Serum INSL3 was measured in 46 samples from 15 girls before, during and after GnRH agonist treatment (Relefact LHRH, Sanofi-Aventis) administered every 4 weeks for CPP. Of the 46 samples, 31 were < LOD. The samples were collected before treatment (baseline, 0 months), during treatment (3 and 12 months) and after end of treatment (36 months). Serum INSL3 was above LOD in six out of 15 girls at baseline and, like other measured hormones, relatively high as compared to normal girls (**Figure 4**). The average concentration of serum INSL3 was 0.009 (0.005) ug/L at baseline (n=15) (**Figure 5**). After 3 months of treatment with GnRH agonist INSL3 decreased to 0.007 (0.003) (n=9) and after 12 months treatment all samples were <LOD (n=14) ug/L. In samples collected after end of treatment (36 months) average serum INSL3 increased to 0.024 (0.024) ug/L (n=8). Serum INSL3 was above LOD in six out of 15 girls at baseline and in 7 out of 8 after cessation of therapy.

## Discussion

We previously developed and validated an MS-based assay with sufficient sensitivity (LOD = 0.03 ug/L, 4.5 pM) for measuring serum INSL3 in pubertal boys and men (normal range 0.5 to 1.5 ug/L) (12). This supported INSL3 as a promising biomarker of testicular Leydig cells in males. Analytical sensitivity is a well-known challenge for measuring serum peptides by LC-MS/MS. Here we utilize a newer MS instrument and achieve a lower limit of detection (0.01 ug/ml (1.5 pM)) for serum INSL3. This technological improvement supports a more frequent use of LC-MS/MS in quantification of peptide hormones in serum and permits us to now explore the pathophysiology of INSL3 in females. We find that serum INSL3 increase in healthy girls with the onset of puberty concomitantly with pubertal activation of the Hypothalamic-pituitary-gonadal (HPG) axis as evidenced by increasing FSH and LH levels. This is in accordance with our previous study of serum INSL3 concentrations in the same samples from healthy peripubertal girls using an in-house immunoassay where an increase during puberty was also observed (11).

Circulating INSL3 concentrations correlated with levels of gonadotropins as well as ovarian hormones and strongest correlations were found between INSL3 and other theca cell products like testosterone and androstenedione. This is in line with our previous findings when evaluating INSL3 by immunoassay (11). The reactivation of the HPG axis during puberty leads to an increase in FSH and LH levels, promoting the growth of ovarian follicles to stages producing inhibin B and steroid hormones (2). In adult women, the primary contributors to circulating INSL3 levels are large antral follicles (17). Consequently, we anticipated a positive correlation between INSL3 levels and gonadotropins, as well as hormones produced by medium and large follicles. The strong correlation between INSL3 and androgen levels support the hypothesis that INSL3 directly stimulates androgen biosynthesis in theca interna cells surrounding large follicles (18). Circulating levels of AMH produced by small ovarian follicles increase prior to pubertal onset (14). This may explain the positive correlation between INSL3 and AMH levels despite being produced from very different stages of ovarian follicles.

We have previously reported that serum INSL3 and other reproductive hormones decrease in men during GnRH agonist treatment (22). In the girls with central precocious puberty (CPP) we also observed a significant decrease in serum INSL3 during treatment with GnRH agonist followed by a marked increase after end of treatment. This is in line with the effect of GnRHa treatment suppressing gonadotropin production and thus arresting maturation of follicles not reaching stages producing INSL3 (as well as other ovarian hormones). Interestingly, we observed a relatively high concentration of serum INSL3 in CPP girls in samples collected at baseline, as compared to healthy girls of the same relatively young age. This supports premature HPG activation and maturation of follicles producing INSL3 in girls with CPP. Due to pulsatile and diurnal variation of reproductive hormone secretion, it is difficult to differentiate between girls with premature thelarche and CPP using unstimulated serum concentration of hormones. After the transient activation of the HPG axis during minipuberty, GnRH testing is therefore implemented as routine clinical practice to distinguish between PT and CPP (23). We have recently reported that none of the established clinical and biochemical parameters were individually adequate as diagnostic markers for CPP vs. premature thelarche; however, a multivariate marker based on several relevant parameters increased the predictive value for CPP (23). Similarly, based on the current study we propose that serum INSL3 may not be a superior marker for CPP when assessed out of context with other parameters. Future studies in a larger group of CPP patients may reveal if adding serum INSL3 to the classical panel of reproductive hormones may be useful in the work-up of girls with suspected early puberty.

The present method measures 5-fold lower levels of serum INSL3 than what has been reported in our previous studies of the same samples using immunoassay (11) and, in addition, there was no significant correlation between the two methods. On the contrary, in a previous direct comparison of the LC-MS/MS and immunoassay methods for serum INSL3 in healthy men there was good correlation between the two methodologies, although slightly higher serum INSL3 levels were obtained with the LC-MS/MS-based method (12). Currently we cannot explain the 5-fold difference and lack of correlation between the two methods when applied to the present female serum samples, but speculate that calibration, matrix effects and inherent differences with the two analytical methods may play a role. Also, there will always be higher imprecision in the lower range for any assay. The measured sample cohort is too small to be useful as a normal range for pubertal girls, but we note that a substantial number of samples from healthy pubertal girls were below the LOD of both the LC-MS/MS and immunoassay. This was particularly true for samples collected before the onset of puberty, but also several samples collected after puberty were below the LOD of the method.

We would like to emphasize the shortcomings of the study. The primary limitation of the study is the relatively small sample sizes of healthy girls and patients with CPP. However, the longitudinal design provides valuable insight into the course of circulating INSL3 according to pubertal development as well as response to inhibition of the HPG axis during GnRHa treatment. Larger studies would clarify if the method is sufficiently sensitive to detect small increases in serum INSL3 in normal puberty in the majority of healthy girls, or possibly only in a small subgroup. As there will always be higher imprecision in the lower range of quantification future studies should be designed to investigate how this may challenge a possibly clinical utility of the method – for example, a future study may do replicate measurements of clinical samples with relatively low serum INSL3.

To conclude, we present a novel LC-MS/MS-based method for assessing serum INSL3 concentrations allowing sensitive quantification of INSL3 in peripubertal girls. The method can be used to further investigate the clinical use of INSL3 in female pediatrics. We note that the present data does not support that INSL3 is a superior marker for precocious puberty as compared to the other hormones measured in this study. Future studies in a larger group of CPP individuals may reveal if serum INSL3 may contribute to the panel of biomarkers for pubertal onset which in combination may contribute to distinguish between premature thelarche and central precocious puberty (23).

## Data availability statement

The original contributions presented in the study are included in the article/supplementary material. Further inquiries can be directed to the corresponding author.

## Ethics statement

The studies involving humans were approved by The Committees on Health Research Ethics in the Capital Region of Denmark. The studies were conducted in accordance with the local legislation and institutional requirements. Written informed consent for participation in this study was provided by the participants’ legal guardians/next of kin.

## Author contributions

JA: Writing – review & editing, Writing – original draft, Methodology, Formal analysis. CH: Writing – review & editing, Writing – original draft, Methodology, Conceptualization. AJ: Writing – review & editing, Writing – original draft, Supervision, Methodology, Data curation, Conceptualization.

## References

[1] Ivell R Anand-Ivell R. Biology of insulin-like factor 3 in human reproduction. Hum Reprod Update. (2009) 15:463–76. doi: 10.1093/humupd/dmp011 19329805

[2] Hagen CP Mouritsen A Mieritz MG Tinggaard J Wohlfart-Veje C Fallentin E. Circulating AMH reflects ovarian morphology by magnetic resonance imaging and 3D ultrasound in 121 healthy girls. Clin Endocrinol Metab. (2015) 100:880–90. doi: 10.1210/jc.2014-3336 25485726

[3] Anand-Ivell R Tremellen K Dai Y Heng K Yoshida M Knight PG. Ivell R Circulating insulin-like factor 3 (INSL3) in healthy and infertile women. Hum Reprod. (2013) 28:3093–102. doi: 10.1093/humrep/det349 24014601

[4] Satchell L Glister C Bleach EC Glencross RG Bicknell AB Dai Y. Knight PG Ovarian expression of insulin-like peptide 3 (INSL3) and its receptor (RXFP2) during development of bovine antral follicles and corpora lutea and measurement of circulating INSL3 levels during synchronized estrous cycles. Endocrinology. (2013) 154:1897–906. doi: 10.1210/en.2012-2232 23546605

[5] Hillier SG Whitelaw PF. Smyth CD Follicular oestrogen synthesis: the 'two-cell, two-gonadotrophin' model revisited. Mol Cell Endocrinol. (1994) 100:51–4. doi: 10.1016/0303-7207(94)90278-X 8056158

[6] Zimmermann S Steding G Emmen JM Brinkmann AO Nayernia K Holstein AF. Adham IM Targeted disruption of the Insl3 gene causes bilateral cryptorchidism. Mol Endocrinol. (1999) 13:681–91. doi: 10.1210/mend.13.5.0272 10319319

[7] Spanel-Borowski K Schäfer I Zimmermann S Engel W. Adham IM Increase in final stages of follicular atresia and premature decay of corpora lutea in Insl3-deficient mice. Mol Reprod Dev. (2001) 58:281–6. doi: 10.1002/(ISSN)1098-2795 11170269

[8] Baerwald AR Adams GP. Pierson RA Ovarian antral folliculogenesis during the human menstrual cycle: a review. Hum Reprod Update. (2012) 18:73–91. doi: 10.1093/humupd/dmr039 22068695

[9] Havelock JC Bay K Ivell R Bathgate RA Rodgers RJ. Carr BR A novel hormone known as Insulin-like factor 3 (INSL3) is expressed in the human ovary and serum levels are increased in women with polycystic ovary syndrome (PCOS). Fertil Steril. (2005) 84:S3–4. doi: 10.1016/j.fertnstert.2005.07.019

[10] Gambineri A Patton L De Iasio R Pallador F Pagotto U. Pasquali R Insulin-like factor 3: a new circulating hormone related to luteinizing hormone-dependent ovarian hyperandrogenism in the polycystic ovary syndrome. J Clin Endocrinol Metab. (2007) 92:2066–73. doi: 10.1210/jc.2006-1678 17356050

[11] Hagen CP Mieritz MG Nielsen JE Anand-Ivell R Ivell R. Juul A Longitudinal assessment of circulating insulin-like peptide 3 levels in healthy peripubertal girls. Fertil Steril. (2015) 103:780–6. doi: 10.1016/j.fertnstert.2014.11.014 25516081

[12] Albrethsen J Frederiksen H Andersson AM Anand-Ivell R Nordkap L Bang AK. Development and validation of a mass spectrometry-based assay for quantification of insulin-like factor 3 in human serum. Clin Chem Lab Med. (2018) 56:1913–20. doi: 10.1515/cclm-2018-0171 29847312

[13] Aksglaede L Sorensen K Petersen JH Skakkebaek NE. Juul A Recent decline in age at breast development: the Copenhagen Puberty Study. Pediatrics. (2009) 123:e932–9. doi: 10.1542/peds.2008-2491 19403485

[14] Hagen CP Aksglaede L Sorensen K Mouritsen A Andersson AM Petersen JH. Juul A Individual serum levels of anti-Mullerian hormone in healthy girls persist through childhood and adolescence: a longitudinal cohort study. Hum Reprod. (2012) 27:861–6. doi: 10.1093/humrep/der435 22215627

[15] Mouritsen A Aksglaede L Soerensen K Hagen CP Petersen JH Main KM. Juul A The pubertal transition in 179 healthy Danish children: associations between pubarche, adrenarche, gonadarche, and body composition. Eur J Endocrinol. (2013) 168:129–36. doi: 10.1530/EJE-12-0191 23093700

[16] Sorensen K Aksglaede L Petersen JH. Juul A Recent changes in pubertal timing in healthy Danish boys: associations with body mass index. J Clin Endocrinol Metab. (2010) 95:263–70. doi: 10.1210/jc.2009-1478 19926714

[17] Irving-Rodgers HF Bathgate RA Ivell R Domagalski R. Rodgers RJ Dynamic changes in the expression of relaxin-like factor (INSL3), cholesterol side-chain cleavage cytochrome p450, and 3beta-hydroxysteroid dehydrogenase in bovine ovarian follicles during growth and atresia. Biol Reprod. (2002) 66:934–43. doi: 10.1095/biolreprod66.4.934 11906911

[18] Glister C Satchell L Bathgate RA Wade JD Dai Y Ivell R. Knight PG Functional link between bone morphogenetic proteins and insulin-like peptide 3 signaling in modulating ovarian androgen production. Proc Natl Acad Sci U.S.A. (2013) 110:E1426–35. doi: 10.1073/pnas.1222216110 PMC3625357 23530236

[19] Sørensen K Mouritsen A Mogensen SS Aksglaede L. Juul A Insulin sensitivity and lipid profiles in girls with central precocious puberty before and during gonadal suppression. J Clin Endocrinol Metab. (2010) 95:3736–44. doi: 10.1210/jc.2010-0731 20484471

[20] Hagen CP Sørensen K Anderson RA. Juul A Serum levels of antimüllerian hormone in early maturing girls before, during, and after suppression with GnRH agonist. Fertil Steril. (2012) 98:1326–30. doi: 10.1016/j.fertnstert.2012.07.1118 22901847

[21] Lynch KL. CLSI C62-A: a new standard for clinical mass spectrometry. Clin Chem. (2016) 62:24–9. doi: 10.1373/clinchem.2015.238626 26430075

[22] Albrethsen J Østergren PB Norup PB Sønksen J Fode M Kistorp C. Juul A serum insulin-like factor 3, testosterone, and LH in experimental and therapeutic testicular suppression. J Clin Endocrinol Metab. (2023) 108:2834–9. doi: 10.1210/clinem/dgad291 37235781

[23] Wang AC Hagen CP Johannsen TH Madsen AG Cleemann LH Christiansen P. Differentiation of idiopathic central precocious puberty from premature thelarche using principal component analysis. J Clin Endocrinol Metab. (2024) 109:370–9. doi: 10.1210/clinem/dgad535 37698163

